# Validation of the Thai version of the quality of recovery scale (QoR-14-Thai) after elective abdominal surgery under general anesthesia

**DOI:** 10.1186/s12871-025-03044-8

**Published:** 2025-04-23

**Authors:** Lalisa Saeaeh, Pornprom Sitthivethayanont, Theerawat Chalacheewa, Tharin Thampongsa, Chakrit Sukying, Rojnarin Komonhirun, Lisa Sangkum

**Affiliations:** 1 270 Department of Anesthesiology, Faculty of Medicine, Ramathibodi hospital, Mahidol University, Rama VI Road, Phaya Thai, Ratchatewi, Bangkok, 10400 Thailand; 2 270 Department of Surgery, Faculty of Medicine, Ramathibodi hospital, Mahidol University, Rama VI Road, Phaya Thai, Ratchatewi, Bangkok, 10400 Thailand; 3 270 Department of Psychiatry, Faculty of Medicine, Ramathibodi hospital, Mahidol University, Rama VI Road, Phaya Thai, Ratchatewi, Bangkok, 10400 Thailand

**Keywords:** Quality of recovery, Postoperative outcomes, Patient-reported outcome measures, Patient outcome assessment

## Abstract

**Background:**

The 15-item Quality of Recovery scale (QoR-15), a short form of the QoR-40, is a widely used self-reported tool for measuring the postoperative quality of recovery. It has been translated into many languages. In this study, we aimed to validate a translated Thai version of the QoR-15 in patients undergoing elective abdominal surgery under general anesthesia.

**Methods:**

This was a single-center observational cohort study. The QoR-15 was translated into Thai and culturally adapted, which led to the items on severe and moderate pain being merged, yielding a 14-item scale: the QoR-14-Thai. Next, the QoR-14-Thai, a checklist measuring the patients’ activities of daily living (ADL), and a 100-mm visual analog scale for assessing their global health (VAS-GH) were administered to the study patients before and 24 h after their abdominal surgery. The validity, reliability, responsiveness, and feasibility of the QoR-14-Thai were assessed.

**Results:**

Among 166 patients, 140 completed the questionnaires, achieving a questionnaire completion rate of 100%. We observed moderate convergent validity between the postoperative QoR-14-Thai and the VAS-GH (*r* = 0.54, *p* < 0.001) and ADL checklist (*r* = 0.50, *p* < 0.001). The QoR-14-Thai was negatively correlated with the length of hospital stay (*r* = − 0.23, *p* < 0.006) and postoperative admission to the intensive care unit (*r* = − 0.85, *p* = 0.001). The QoR-14-Thai had excellent internal consistency (Cronbach’s alpha = 0.869), split-half reliability (0.913), test–retest reliability (0.94), and high responsiveness (Cohen’s effect size: 1.01, standardized response mean: 0.73). The median time to complete the questionnaire was 2 min (interquartile range: 1–2).

**Conclusions:**

The QoR-14-Thai was deemed a valid, reliable, and convenient tool for evaluating the quality of recovery after elective abdominal surgery.

**Trial registration:**

This study was registered prospectively on the Thai Clinical Trials Registry, identifier TCTR20210326009, on March 26, 2021.

**Supplementary Information:**

The online version contains supplementary material available at 10.1186/s12871-025-03044-8.

## Introduction

Postoperative recovery is a complex and multidimensional process involving physical, physiological, psychological, and economic components [1]. Most studies assessing postoperative recovery have focused on measuring physical endpoints (e.g., pain score and bowel function) or the incidence of adverse events (e.g., morbidity and mortality rate). Although these parameters are important, the quality of recovery from the patient’s perspective is often neglected. The use of patient-reported outcomes (PROs) during the perioperative period can provide a more comprehensive view of postoperative recovery. A widely used tool for evaluating PROs is the quality of recovery (QoR).

The 40-item QoR scale (QoR-40) was developed by Myles et al. [2] and has been translated and validated into Thai as the Thai QoR-35 [3]. Its validity, reliability, responsiveness, and feasibility in measuring postoperative health status and anesthesia have been demonstrated in different patient groups [4]. The QoR-15 was first developed and evaluated for surgical patients undergoing surgery and general anesthesia [5]. It is a short form of the QoR-40. Stark et al. stated that the QoR-15 had psychometric properties comparable to the QoR-40 and was acceptable by the patients [5]. The QoR-15 has been translated and validated in several languages [6, 7, 8, 9, 10, 11, 12]. A comprehensive scoping review conducted in 2010 utilized the “consensus-based standards for the selection of health measurement instruments” (COSMIN) criteria to assess the quality of patient-reported outcome measures used for evaluating postoperative recovery [13]. Overall, the QoR-15 met the COSMIN standards for patient-reported outcome measurement and has been reported as a valid, reliable, and responsive patient-centered outcome metric for surgical patients [14]. Moreover, the European Society of Anesthesiology and the American Society for Enhanced Recovery and Perioperative Quality Initiative recommended the QoR-15 for evaluating perioperative clinical outcome [15].

In this study, we translated and validated the Thai version of the QoR-15 and analyzed its validity, reliability, responsiveness, and clinical feasibility in patients undergoing elective abdominal surgery.

## Materials and methods

### Study design

This prospective observational study was approved by the human research ethics committee of Ramathibodi Hospital, Mahidol University, Bangkok, Thailand (ID MURA2021/29) and was registered prospectively on the Thai Clinical Trials Registry (TCTR20210326009) on March 26, 2021. Participant enrollment commenced on April 5, 2021, and concluded in February 2022.

All patients provided written informed consent prior to participation. The study was conducted in accordance with the Declaration of Helsinki and followed the Strengthening the Reporting of Observational studies in Epidemiology (STROBE) guidelines [16].

### Patient population

We included patients who were over 18 years of age, classified as American Society of Anesthesiologists (ASA) class I–III, scheduled to undergo elective open or laparoscopic abdominal surgery under general anesthesia (including gynecological, urological, and general surgeries), and capable of completing the questionnaires. Patients were excluded if they refused to provide consent, were unable to read Thai, had cognitive impairments, had a history of alcohol or drug dependence, or had severe preexisting medical conditions that limited postoperative assessment.

### Translation and cultural adaption of the QoR-15

After receiving permission from the authors of QoR-40 [5] and QoR-35 Thai [3], we performed the following three-step process to translate the original English version of the QoR-15 into Thai according to recommendations [17] and previous validation studies [10, 11]. First, the questionnaire was critically reviewed for appropriateness and suitability by a committee comprising three anesthesiologists, one surgeon, and one psychiatrist. Each item was evaluated using a three-point scoring system (− 1, 0, 1), with 1 indicating absolute agreement and − 1 indicating absolute disagreement. Items with > 0.5 points were considered to have good agreement and were retained. The remaining items were considered inappropriate and reconsidered. Upon review, the items of “severe pain” and “moderate pain” were considered too confusing for the patients. Therefore, these two items were combined, yielding a total of 14 items. Next, two experienced anesthesiologists (L.S. and L.S.), who are proficient in both languages, translated the QoR-15 into Thai by using the QoR-35 Thai as reference [3]. Second, the translated version was back translated into English by one English linguistic academician. Finally, each question was rendered in its most comprehensive form by the aforementioned committee. The QoR-14-Thai used in this study is available in supplementary file 1)

We then conducted a pilot survey to administer the QoR-14-Thai to randomly 20 patients who were not included in the study. The questionnaire did not require any further modifications and was finalized.

### Study protocol

The day before their surgery, informed consent was obtained, and the patients were asked to complete three questionnaires to evaluate their baseline status: the QoR-14-Thai, a checklist assessing the activities of daily living (ADLs), and a 100-mm visual analog scale for assessing their global health (VAS-GH). The patients were asked to complete these three questionnaires again 24 h after surgery to assess their postoperative recovery status.

The QoR-14-Thai includes five domains of recovery outcomes: physical comfort, emotional state, physical independence, psychological support, and pain. The minimum score of 0 (very poor recovery) and a maximum score of 140 (excellent recovery).

The 100-mm VAS-GH measures the patient’s global health, ranging from 0 = “worst health status” and 100 = “best health status” [5]. The ADL checklist is a simple tool for self-evaluation of independence, encompassing six basic activities: bathing, dressing, toileting, transferring, continence, and feeding. The minimum and maximum scores for the ADL assessment are 6 and 12, respectively (supplementary file 2). We assessed the ADLs because these are associated with clinical outcomes in elective major abdominal surgery [18], hip fractures [19], and geriatric trauma [20]. Additionally, we collected the patients’ basic clinicodemographic information, including age, sex, weight, height, ASA class, level of education, underlying conditions, type of surgery, and perioperative complications.

### Statistical analysis

#### Sample size estimations

The sample size was calculated using a power of 80% and a type I error rate of 0.05. It is recommended to have a sample size of 10 patients per item to validate a questionnaire [21, 22]. Therefore, a minimum sample size of 140 was selected to ensure sufficient power for evaluating the hypothesis.

### Data analysis

Data are presented as mean ± standard deviation (SD), median (interquartile range [IQR]), or number (percentage) where appropriate. Associations were measured using Pearson’s correlation coefficient (r) or Spearman’s rho (ρ). Internal consistency was measured using Cronbach’s alpha. Test–retest reliability was measured using the intraclass correlation coefficient. All statistical analyses were performed using SPSS v18.0 (SPSS, Chicago, IL, USA). *P* < 0.05 was set as significant.

### Psychometric evaluation

The psychometric assessment of the QoR-14-Thai was conducted as described in the original study [5] and subsequent studies [6, 7, 8, 9, 10, 11, 12].

### Construct validity

Construct validity was evaluated through both convergent and discriminant validity. Convergent validity was measured by analyzing the correlation between the QoR-14-Thai with VAS-GH and the ADL checklist. Additionally, the QoR-14-Thai score was further tested with the hypothesis that it would have a negative association with ASA physical status, operative time, blood loss, perioperative complications, postoperative admission to the intensive care unit (ICU), and length of hospital stay.

Discrimination validity was measured by comparing the scores of QoR-14-Thai with patients with complications and the VAS-GH of ≥ 70 or < 70 mm [5].

The floor and ceiling effects of the QoR-14-Thai were assessed by evaluating whether < 15% respondents achieved the highest (100) or lowest (0) possible scores [23].

### Reliability

Reliability was assessed using internal consistency (Cronbach’s alpha) and split-half reliability of the QoR-14-Thai. The correlation between two segments of the QoR-14-Thai was also analyzed. Test–retest reliability was evaluated by repeating the QoR-14-Thai in a subset of 70 patients within 30–60 min. The correlation between measurements was then assessed.

### Responsiveness

Responsiveness describes an instrument’s sensitivity or ability to detect clinically important change. This was quantified using Cohen’s effect size and standardized response mean.

### Acceptability and feasibility

Acceptability and feasibility were measured using the time taken for patients to complete the questionnaire, recruitment, and successful completion rate.

## Results

### Demographic data

**Fig. 1:**
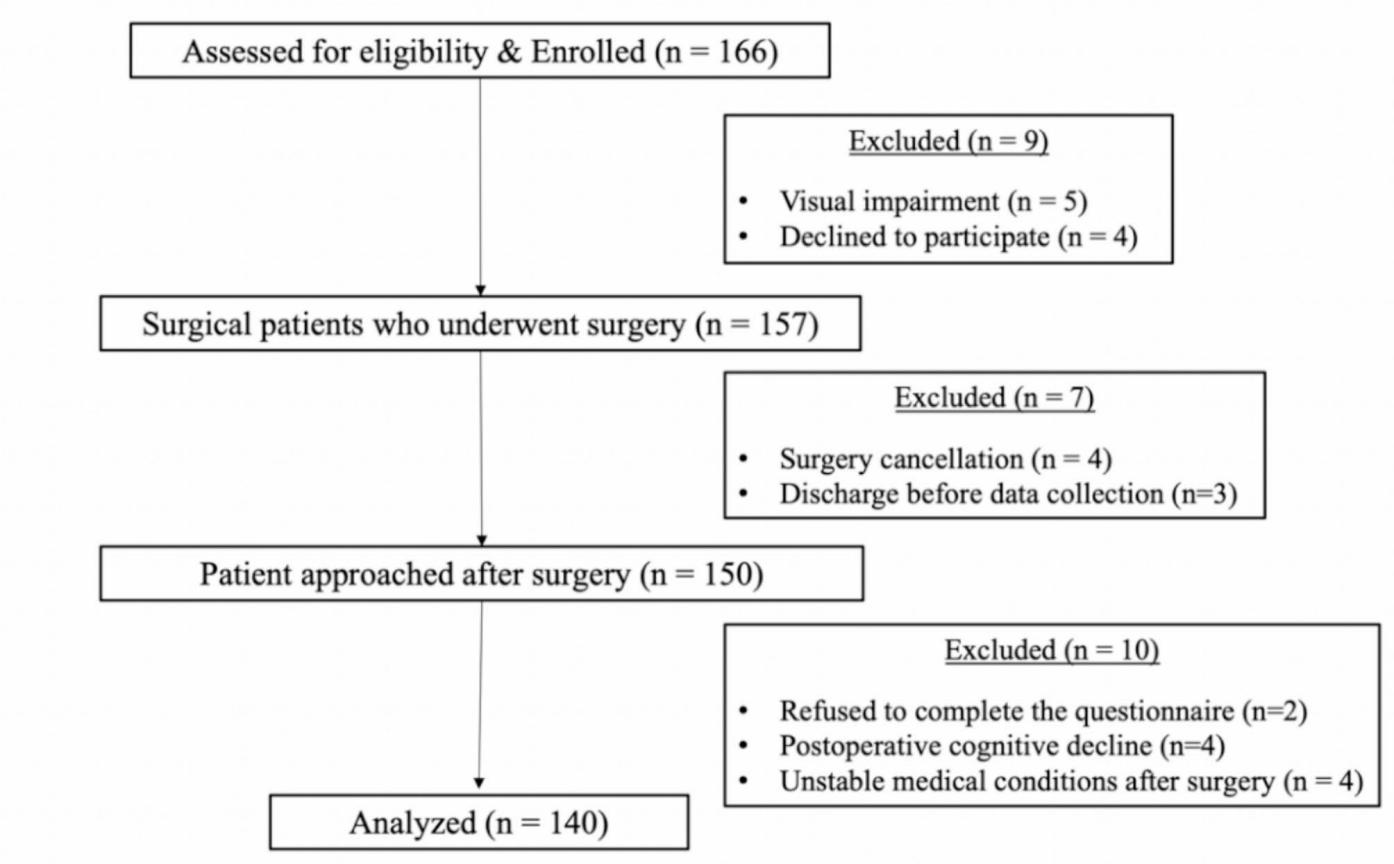
Study flowchart

From April 2021 to February 2022, we recruited 166 patients. Of these, four declined to participate, five had visual impairments, four had their surgeries canceled, three were discharged before data collection, and ten were unable to complete the questionnaire. After all, 140 patients were included in our study (Fig. 1).

Table 1 summarizes the patients’ clinicodemographic characteristics. The mean age of the patients was 51 years. More than 50% of the patients had ASA II or III and underwent gynecological surgery. The median duration of surgery was 227.5 min. The median length of hospital stay was 4 days.

**Table 1:** Patient’s clinicodemographic characteristics (*n* = 140)

Variables	*n* = 140
Age (years), mean (SD)	51.27 (14.8)
Gender, n (%)	
- Male	48 (34.29)
- Female	92 (65.71)
Body mass index (kg.m2), mean (SD)	25.17 (4.67)
ASA PS, n (%)	
- I	33 (23.57)
- II	79 (56.43)
- III	28 (20)
Education, n (%)	
- Less than high school	25 (17.85)
- High school	27 (19.28)
- Bachelor’s degree	67 (47.85)
- Master’s degree	19 (13.57)
- Others	2 (1.45)
History of previous surgery, n (%)	87 (62.14)
Preexisting medical conditions, n (%)	
- Neurological	11 (6.75)
- Cardiovascular	57 (34.95)
- Respiratory	8 (4.91)
- Renal	11 (6.75)
- Endocrinological	59 (36.20)
- Other	17 (12.14)
Type of surgery, n (%)	
- General surgery	48 (34.29)
- Urological surgery	22 (15.71)
- Gynecological surgery	70 (50.00)
Anesthesia technique, n (%)	
- General anesthesia	136 (97.14)
- Total intravenous anesthesia	1 (0.72)
- Combined GA–RA	3 (2.14)
Duration of surgery (mins), median (IQR)	227.5 (167.5–307.5)
Blood loss (ml), median (IQR)	100 (40–300)
Intraoperative complications, n (%)	
- Anemia	1 (0.71)
- Bradycardia	8 (5.71)
- Bronchospasm	1 (0.71)
- Delayed emergences need treatment	1 (0.71)
- Others	5 (3.57)
Postoperative admission to the intensive care unit, n (%)	11 (7.86)
Length of hospital stay (days), median (IQR)	4 (3–6)

Data are represented as mean ± standard deviation, median (interquartile range), or n (%), where appropriate. *ASA* American Society of Anesthesiologists, *GA* General anesthesia, *RA* Regional anesthesia

### Recovery scores

**Fig. 2:**
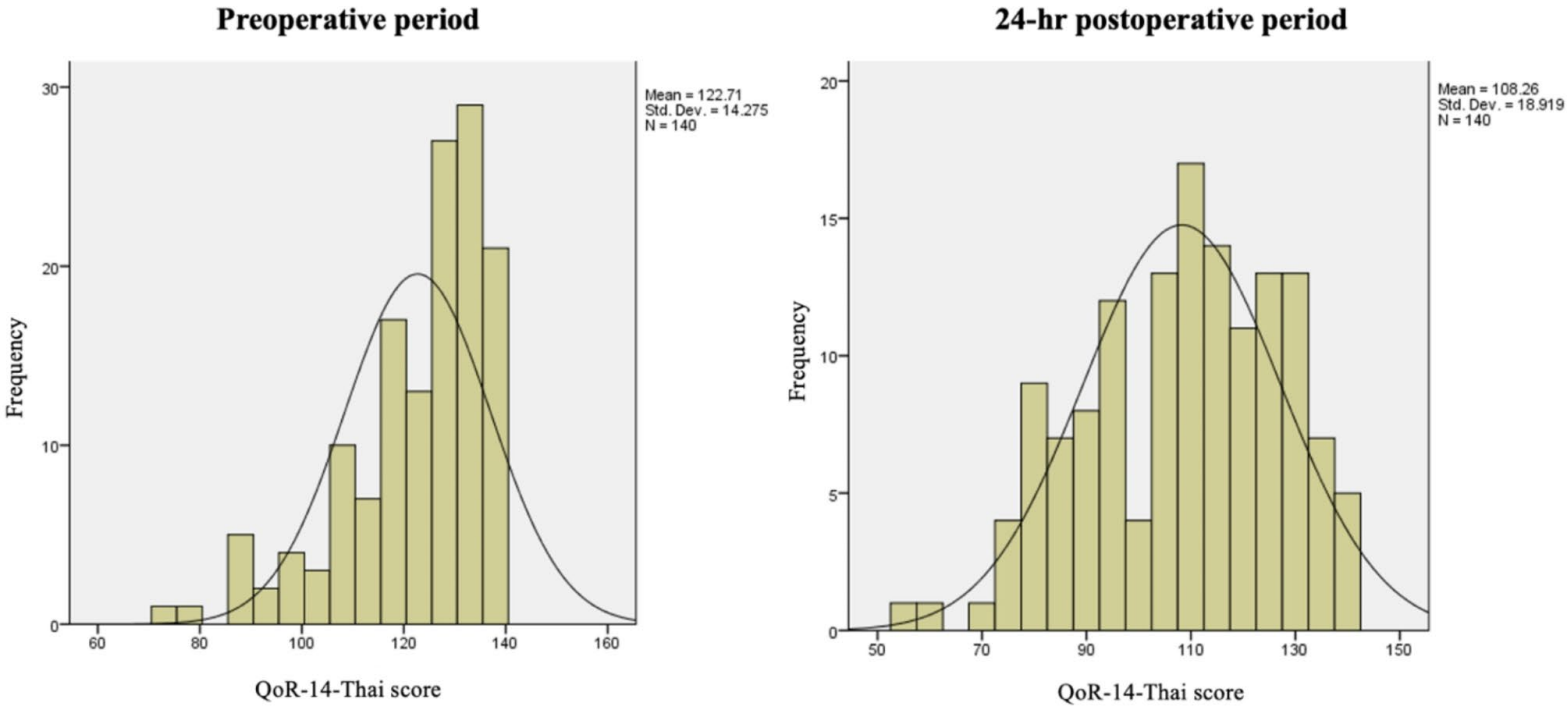
Histogram of the QoR-14-Thai scores at the preoperative and 24-hour postoperative time points

The scores of the three questionnaires (QoR-14-Thai, ADL, and VAS-GH) are presented in Table 2. The postoperative scores were significantly lower than the baseline values. The mean QoR-14-Thai scores in the preoperative and postoperative periods were 122.71 ± 14.27 and 108.26 ± 18.92, respectively; the mean difference was − 14.46 (− 11.14 to − 17.78). The distribution of 140 postoperative QoR-14-Thai is presented in Fig. 2.

**Table 2:** Preoperative and 24-h postoperative scores of the QoR-14-Thai, ADL, and VAS-GH questionnaires

	Preoperative	24-h postoperative	*p*-value
QoR-14-Thai	122.71 ± 14.27	108.26 ± 18.92	< 0.001
VAS-GH	89.75 ± 9.99	77.89 ± 17.31	< 0.001
ADL	5.97 ± 0.27	4.66 ± 1.79	< 0.001

Data are represented as mean ± standard deviation

### Validity

Convergent validity assessment revealed moderate correlations between the QoR-14-Thai and VAS-GH (*r* = 0.54, *p* < 0.001) and between the QoR-14-Thai and ADL (*r* = 0.51, *p* < 0.001; Table 3). The postoperative QoR-14-Thai scores were negatively correlated with the length of hospital stay (*r* = − 0.23, p value = 0.006) and postoperative admission to the ICU (*r* = − 0.85, *p* = 0.001). No significant associations were noted between the postoperative QoR-14-Thai score and patient age (*p* = 0.22), ASA physical status (*p* = 0.89), blood loss (*p* = 0.27), and duration of surgery (*p* = 0.123). There was no difference in the postoperative QoR-14-Thai scores between men and women (*p* = 0.27).

**Table 3:** Correlation coefficients of the QoR-14-Thai with ADL and VAS-GH

Correlation coefficients		
QoR-14-Thai	ADL	VAS-GH
Baseline	0.21 (*p* = 0.011)	0.37 (*p* < 0.001)
24-hour after surgery	0.51 (*p* < 0.001)	0.54 (*p* < 0.001)

The QoR-14-Thai score was significantly different between patients with good and poor global health (112.31 ± 17.41 vs. 91.3 ± 15.42, *p* < 0.001). However, patients who experienced postoperative complications had comparable postoperative QoR-14-Thai scores (109.75 ± 20.82 vs. 108.06 ± 18.74, p 0.74).

Ceiling and flooring effects were within the acceptable limit (< 15%), with no significant flooring or ceiling effect.

### Reliability

The interitem and dimension correlation matrices of the postoperative QoR-14-Thai scores are presented in Table 4. It had high reliability (Cronbach’s alpha: 0.87, split-half reliability: 0.91, test–retest reliability: 0.94). Reproducibility was considered good.

**Table 4:** Interitem correlation matrix for the postoperative QoR–14-Thai scores by item

QoR-14 item No.	VAS after	Total	1	2	3	4	5	6	7	8	9	10	11	12	13
1	0.376	0.634	1												
*p-value*	*< 0.001*	*< 0.001*	*-*												
2	0.493	0.694	0.431	1											
*p-value*	*< 0.001*	*< 0.001*	*< 0.001*	*-*											
3	0.298	0.605	0.531	0.394	1										
*p-value*	*< 0.001*	*< 0.001*	*< 0.001*	*< 0.001*	*-*										
4	0.217	0.625	0.412	0.329	0.709	1									
*p-value*	*0.010*	*< 0.001*	*< 0.001*	*< 0.001*	*< 0.001*	*-*									
5	0.510	0.787	0.513	0.598	0.589	0.554	1								
*p-value*	*< 0.001*	*< 0.001*	*< 0.001*	*< 0.001*	*< 0.001*	*< 0.001*	*-*								
6	0.470	0.764	0.420	0.597	0.337	0.348	0.530	1							
*p-value*	*< 0.001*	*< 0.001*	*< 0.001*	*< 0.001*	*< 0.001*	*< 0.001*	*< 0.001*	*-*							
7	0.536	0.737	0.350	0.596	0.283	0.256	0.627	0.758	1						
*p-value*	*< 0.001*	*< 0.001*	*< 0.001*	*< 0.001*	*0.001*	*0.002*	*< 0.001*	*< 0.001*	*-*						
8	0.371	0.702	0.524	0.437	0.414	0.500	0.510	0.606	0.488	1					
*p-value*	*< 0.001*	*< 0.001*	*< 0.001*	*< 0.001*	*< 0.001*	*< 0.001*	*< 0.001*	*< 0.001*	*< 0.001*	*-*					
9	0.164	0.487	0.612	0.303	0.354	0.361	0.334	0.279	0.235	0.587	1				
*p-value*	*0.053*	*< 0.001*	*< 0.001*	*< 0.001*	*< 0.001*	*< 0.001*	*< 0.001*	*0.001*	*0.005*	*< 0.001*	*-*				
10	0.425	0.709	0.582	0.376	0.472	0.496	0.621	0.462	0.457	0.626	0.675	1			
*p-value*	*< 0.001*	*< 0.001*	*< 0.001*	*< 0.001*	*< 0.001*	*< 0.001*	*< 0.001*	*< 0.001*	*< 0.001*	*< 0.001*	*< 0.001*	*-*			
11	0.216	0.535	0.203	0.262	0.179	0.296	0.355	0.232	0.292	0.223	0.056	0.230	1		
*p-value*	*0.010*	*< 0.001*	*0.016*	*0.002*	*0.034*	*< 0.001*	*< 0.001*	*0.006*	*< 0.001*	*0.008*	*0.514*	*0.006*	*-*		
12	0.020	0.429	0.123	0.202	0.185	0.144	0.170	0.217	0.189	0.060	0.031	0.161	0.386	1	
*p-value*	*0.810*	*< 0.001*	*0.147*	*0.017*	*0.028*	*0.090*	*0.045*	*0.010*	*0.025*	*0.481*	*0.714*	*0.057*	*< 0.001*	*-*	
13	0.319	0.539	0.141	0.208	0.122	0.225	0.302	0.288	0.286	0.226	0.058	0.264	0.460	0.293	1
*p-value*	*< 0.001*	*< 0.001*	*0.096*	*0.014*	*0.150*	*0.008*	*< 0.001*	*0.001*	*0.001*	*0.007*	*0.496*	*0.002*	*< 0.001*	*< 0.001*	*-*
14	0.173	0.403	0.165	0.075	0.056	0.160	0.151	0.226	0.124	0.171	0.069	0.182	0.256	0.289	0.565
*p-value*	*0.041*	*< 0.001*	*0.052*	*0.380*	*0.508*	*0.059*	*0.075*	*0.007*	*0.143*	*0.043*	*0.416*	*0.032*	*0.002*	*0.001*	*< 0.001*

* *p* < 0.05 (two-tailed)

### Responsiveness

Responsiveness indicators revealed excellent values (Cohen effect size: 1.01, standardized response mean: 0.73), implying that the QoR-14-Thai can detect changes in the quality of recovery and that the quality of the original version is preserved. The changes in each perioperative health status are summarized in Table 5.

**Table 5:** Changes in the QoR-14-Thai of the participants 24 h after surgery

QoR-14 Item	Preoperative	Postoperative	Mean Change (95%CI)	%Change from Baseline	Cohen Effect Size	Standardized Response Mean
1. Able to breathe easy	9.25 ± 1.27	8.69 ± 1.56	-0.56 (-0.3 to -0.83)	6	0.44	0.35
2. Been able to enjoy food	8.16 ± 1.86	6.36 ± 2.56	-1.8 (-1.35 to -2.25)	22	0.97	0.66
3. Feeling rested	8.28 ± 1.56	7.96 ± 1.8	-0.32 (0.01 to -0.66)	4	0.21	0.16
4. Have had a good sleep	7.96 ± 1.79	7.82 ± 1.88	-0.14 (0.2 to -0.49)	2	0.08	0.07
5. Having a feeling of general well-being	8.64 ± 1.69	7.02 ± 2.22	-1.61 (-1.21 to -2.01)	19	0.95	0.67
6. Able to look after personal toilet and hygiene unaided	9.73 ± 0.78	7.19 ± 2.9	-2.54 (-2.05 to -3.02)	26	3.27	0.88
7. Able to return to work or usual home activities	9.43 ± 1.43	5.89 ± 3.2	-3.54 (-2.99 to -4.08)	38	2.48	1.08
8. Able to communicate with family or friends	9.6 ± 1.24	8.75 ± 1.93	-0.85 (-0.52 to -1.18)	9	0.69	0.43
9. Getting support from hospital doctors and nurses	9.68 ± 0.79	9.54 ± 1.14	-0.14 (0.04 to -0.32)	1	0.18	0.13
10. Feeling comfortable and in control	9.11 ± 1.28	8.72 ± 1.64	-0.39 (-0.1 to -0.67)	4	0.30	0.23
11. Pain	8.17 ± 2.68	5.65 ± 2.33	-2.52 (-1.97 to -3.08)	31	0.94	0.76
12. Nausea or vomiting	9.31 ± 2.06	8.21 ± 2.57	-1.09 (-0.54 to -1.65)	12	0.53	0.33
13. Feeling worried or anxious	6.41 ± 3.03	7.44 ± 2.54	1.04 (1.52 to 0.55)	16	0.34	0.36
14. Feeling sad or depressed	8.99 ± 2.18	9 ± 1.91	0.01 (0.42 to -0.41)	0	0.00	0.00
Total	122.71 ± 14.27	108.26 ± 18.92	-14.46 (-11.14 to -17.78)	12	1.01	0.73

All data are presented as mean ± SD, unless otherwise stated. Cohen effect size = mean change in score divided by the baseline (preoperative) SD; standardized response mean = mean change in score divided by its SD; QoR = quality of recovery

### Acceptability and feasibility

The median time to completing the QoR-14-Thai was 2 min (IQR: 1–2) before and 2 min (IQR: 1–3) after the surgery. No patient reported a zero score. Six patients before surgery and one after surgery reported the maximum score of 140.

## Discussion

Our study demonstrated that the QoR-14-Thai has acceptable validity, reliability, and clinical feasibility in the Thai population undergoing elective abdominal surgery. The QoR-14-Thai had a significant negative correlation with length of hospital and postoperative admission to the ICU. The internal consistency was excellent, with a high degree of responsiveness and clinical feasibility.

The QoR-15 has been demonstrated to have good to moderate correlation with VAS-GH scores: original English, 0.68; Chinese, 0.63 [12]; Korean, 0.61 [10]; French, 0.6 [7]; Dutch, 0.59 [8]; and German, 0.58 [24]). Consistently, the QoR-14-Thai had a moderate correlation with VAS-GH scores (*r* = 0.54, *p* < 0.001).

The QoR-14-Thai was also superior to the ADL checklist for evaluating global health status after surgery. The Cohen effect size and standardized response mean of the QoR-14-Thai were 1.01 and 0.73, respectively, indicating moderate to high responsiveness [25]. Internal consistency was excellent, as measured using Cronbach’s alpha and split-half reliability (both > 0.80) [26]. Compared to previous studies, our results demonstrated that the QoR-14-Thai score was negatively correlated with the length of hospital stay and postoperative admission to the ICU. However, the QoR-14-Thai score did not correlate with patient age, ASA PS class, or duration of surgery. While our findings aligned with previous studies, certain factors, such as age [8], ASA PS class [27], and duration of surgery [5, 10, 27], did not show a correlation with the QoR-14-Thai score. We hypothesize that this discrepancy may be due to differences in the study population and types of surgery. Specifically, our participants were younger, categorized as ASA PS class I or II, and primarily underwent gynecologic procedures, with no major surgeries included. Moreover, the QoR-14-Thai failed to distinguish patients who experienced perioperative complications. We hypothesize that the low complication rate in our study may have resulted in insufficient power to detect significant differences in scores. Overall, these results suggest that the QoR-14-Thai is a useful tool for measuring the quality of recovery after elective abdominal surgery in a Thai population.

The QoR-40 (or the Thai QoR-35) may be inconvenient during the perioperative period because the longer time taken to complete the larger questionnaire reduces acceptability and feasibility for the patient and cause an excessive burden to the staff, thus disrupting clinical care. The correlation between Thai QoR-35 and VAS-recovery at 24 h after operation was 0.84 [3]. However, that study included inpatients and outpatients undergoing all types of surgery, and the questionnaire was validated with a 100-mm VAS of recovery status. However, we used VAS-GH, precluding a head-to-head comparison of the two results. The Thai QoR-35 took a mean of 5 min to complete, compared with 2 min for the QoR-14-Thai. This may have contributed to the high rate of participation and successful completion in our study, which highlights the clinical usefulness of the QoR-14-Thai.

The QoR-15 serves as a valuable outcome measure in perioperative clinical trials and for assessing the impact of changes in health care delivery for quality assurance purposes. Therefore, the Enhanced Recovery After Surgery care protocol includes the use of the QoR-15 for monitoring PROs during the perioperative period [15]. The QoR-15 is also sensitive in detecting clinically important differences in postoperative recovery. Perioperative interventions that result in a change of 0.9 for the QoR score, 8 for QoR-15, or 6.3 for QoR-40 signify a clinically important improvement or deterioration [28]. In 2021, the minimal clinically important difference for the QoR-15 was updated to 6.0 [29]. However, we did not assess the minimal clinically important difference in the QoR-14-Thai.

This study has several limitations. First, the QoR-14-Thai was translated and validated in a single tertiary referral center, which may limit the generalizability of our findings, particularly in institutions with fewer high-risk patients. Second, all the patients completed the questionnaires on their own. Therefore, the results may not be generalizable to those who are unable to complete the questionnaire independently. Third, we measured the QoR-14-Thai at 24 h after surgery but not beyond that. Future studies should consider the immediate and late recovery periods. Moreover, we excluded patients with severe medical conditions after surgery that limited postoperative assessment. Fourth, we did not compare the validity of the test with full version (Thai QoR-35). Finally, the clinically important differences were not assessed, necessitating further research to elucidate the minimal clinically important difference in the Thai population.

## Conclusion

The QoR-14-Thai was found to have acceptable validity, reliability, responsiveness, and clinical feasibility for assessing postoperative recovery in a Thai population undergoing elective abdominal surgery under general anesthesia. Despite only moderate correlations with ADL and VAS-GH scores, the QoR-14-Thai had excellent discriminant validity and reliability. Future studies should evaluate the QoR-14-Thai in different settings (e.g., emergency and outpatient settings) as well as the minimal clinically important difference in the Thai population.

## Electronic supplementary material

Below is the link to the electronic supplementary material.

Supplementary Material 1

Supplementary Material 2

## Data Availability

The datasets during and/or analyzed during the current study available from the corresponding author on reasonable request.

## Abbreviations

ADLs: activities of daily living
ASA PS status: American Society of Anesthesiologists physical status
QoR: Quality of Recovery score
PROs: patient-reported outcomes
SD: Standard deviation
VAS-GH: visual analog scale of global health
