# Supplementary material for: Validation of the Thai version of the quality of recovery scale (QoR-14-Thai) after elective abdominal surgery under general anesthesia
Source: BMC Anesthesiol. 2025 Apr 23;25:205. doi: 10.1186/s12871-025-03044-8 (PMC12016262; doi:10.1186/s12871-025-03044-8)
Supplement: Supplementary file 1 — Supplementary Material 1 [file 12871_2025_3044_MOESM1_ESM.pdf]

วันที่ทำการทดสอบ ____/ _____/ ______ ทำแบบสอบถาม ครั้งที่_________

แบบสอบถามคุณภาพการฟื้นจากการระงับความรู้สึก (The Thai QoR-14)

แบบสอบถามนี้จัดทำขึ้นเพื่อใช้ประเมินคุณภาพการฟื้นตัวภายหลังการผ่าตัดของท่าน
คำถามทั้งหมดมี 14 ข้อ ขอให้ท่านตอบแบบสอบถามตรงกับความรู้สึกที่เกิดขึ้นจริงในขณะนั้นและขอให้ท่านตอบคำถามทุกข้อ
เพื่อเป็นประโยชน์แก่การศึกษาและการพัฒนาคุณภาพในการดูแลรักษาผู้ป่วยต่อไป

		ไม่เลย					ปานกลาง					มากที่สุด
1	ฉันหายใจได้สะดวก	0	1	2	3	4	5	6	7	8	9	10
2	ฉันรู้สึกเจริญอาหาร	0	1	2	3	4	5	6	7	8	9	10
3	ฉันรู้สึกได้รับการพักผ่อน	0	1	2	3	4	5	6	7	8	9	10
4	ฉันนอนหลับได้ดี	0	1	2	3	4	5	6	7	8	9	10
5	ฉันรู้สึกเป็นปกติ	0	1	2	3	4	5	6	7	8	9	10
6	ฉันสามารถทำความสะอาดตนเอง เช่น แปรงฟัน ล้างหน้า เข้าห้องน้ำด้วยตนเองได้ (กรณีใช้ไม้ค้ำยัน หากสามารถทำได้ด้วยตนเอง ถือว่าท่านมีความสามารถในข้อนี้)	0	1	2	3	4	5	6	7	8	9	10
7	ฉันสามารถทำกิจวัตรประจำวันตามปกติได้ เช่น ทำงานบ้าน ทำอาหาร (กรณีใช้ไม้ค้ำยัน หากสามารถทำได้ด้วยตนเอง ถือว่าท่านมีความสามารถในข้อนี้)	0	1	2	3	4	5	6	7	8	9	10
8	ฉันสามารถติดต่อกับเพื่อนหรือญาติได้	0	1	2	3	4	5	6	7	8	9	10
9	ฉันได้รับความดูแลช่วยเหลือจากทีมแพทย์และพยาบาล	0	1	2	3	4	5	6	7	8	9	10
10	ฉันรู้สึกสบายและพร้อมที่จะรับมือกับสถานการณ์ต่างๆที่จะเกิดขึ้นภายหลังการผ่าตัด	0	1	2	3	4	5	6	7	8	9	10
		ไม่เลย					ปานกลาง					มากที่สุด
11	ฉันมีคะแนนความปวด	0	1	2	3	4	5	6	7	8	9	10
12	ฉันมีอาการคลื่นไส้ อาเจียน	0	1	2	3	4	5	6	7	8	9	10
13	ฉันรู้สึกวิตกกังวล	0	1	2	3	4	5	6	7	8	9	10
14	ฉันรู้สึกซึมเศร้า	0	1	2	3	4	5	6	7	8	9	10

ใช้เวลาในการทำแบบสอบถามนาน................นาที ได้คะแนน........................ / 140
