# Supplementary material for: Validation of the Thai version of the quality of recovery scale (QoR-14-Thai) after elective abdominal surgery under general anesthesia
Source: BMC Anesthesiol. 2025 Apr 23;25:205. doi: 10.1186/s12871-025-03044-8 (PMC12016262; doi:10.1186/s12871-025-03044-8)

วันที่ทำการทดสอบ ____/ _____/ ______ ทำแบบสอบถาม ครั้งที่__________

แบบประเมินความสามารถในการประกอบกิจวัตรประจำวัน (Katz Activity of daily living)

กรุณา X บน ☐ ที่ท่านมีความสามารถตามหัวข้อที่ประเมิน ดังต่อไปนี้

กิจวัตรประจำวัน	ความสามารถของท่าน
**การอาบน้ำ**	☐ อาบน้ำได้ด้วยตนเอง หรือ มีคนช่วยเหลือไม่เกิน 1 บริเวณ เช่น บริเวณหลัง เป็นต้น<br>☐ ไม่สามารถอาบน้ำด้วยตนเอง ต้องมีคนช่วยหรืออาบน้ำให้
**การสวมใส่เสื้อผ้า**	☐ สามารถหยิบเสื้อผ้าจากที่เก็บและแต่งตัวได้ด้วยตนเองทั้งหมด<br>☐ ไม่สามารถแต่งกายด้วยตนเองทั้งหมด
**การใช้ห้องน้ำ**	☐ สามารถเดินเข้าห้องน้ำ ทำความสะอาด สวมใส่กางเกง/กระโปรง ภายหลังเสร็จกิจธุระได้ด้วยตนเองทั้งหมด<br>☐ ไม่สามารถเข้าห้องน้ำเองได้ ต้องการความช่วยเหลือ เช่น ช่วยในการเดินเข้าห้องน้ำ ทำความสะอาดหรือสวมเครื่องแต่งกาย
**การลุกนั่งจากที่นอน หรือ จากเตียงไปยังเก้าอี้**	☐ สามารถลุกจากเตียง หรือ เก้าอี้ได้ด้วยตนเอง (กรณีใช้อุปกรณ์ช่วยพยุง หากสามารถทำได้ด้วยตนเอง ถือว่าท่านมีความสามารถในข้อนี้)<br>☐ ไม่สามารถลุกจากเตียง หรือ เก้าอี้ได้ด้วยตนเอง
**การควบคุมการขับถ่าย**	☐ สามารถควบคุม/ กลั้น ปัสสาวะและอุจจาระได้<br>☐ ไม่สามารถควบคุม/กลั้น การถ่ายปัสสาวะหรืออุจจาระเองได้
**การรับประทานอาหาร (เมื่อเตรียมสำรับเรียบร้อยไว้ต่อหน้า)**	☐ สามารถรับประทานอาหารได้ด้วยตนเอง<br>☐ ไม่สามารถรับประทานอาหารด้วยตนเอง ต้องมีผู้ให้การช่วยเหลือในการรับประทานอาหาร

ประเมินคะแนนสุขภาพโดยรวม

โปรดทำเครื่องหมาย X บนหมายเลขที่ท่านให้คะแนน เพื่อประเมินสุขภาพโดยรวมของท่านในช่วง 24 ชั่วโมงที่ผ่านมา
โดย 0 = สุขภาพโดยรวมแย่มาก และ 100 = สุขภาพโดยรวมดีมาก

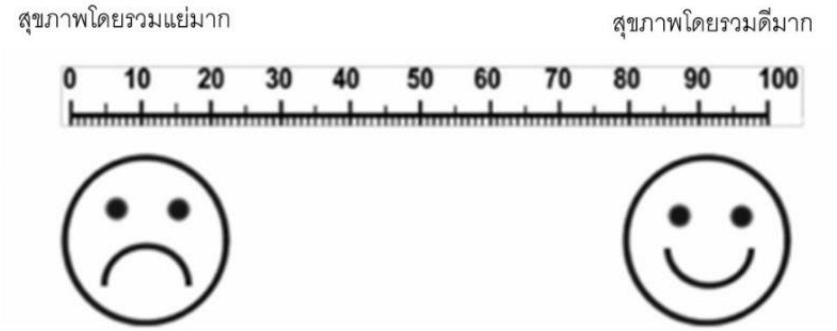

Supplement: Supplementary file 2 — Supplementary Material 2 [file 12871_2025_3044_MOESM2_ESM.pdf]
